# Ethnic differences in colon cancer care in the Netherlands: a nationwide registry-based study

**DOI:** 10.1186/s12885-017-3241-5

**Published:** 2017-05-04

**Authors:** M. Lamkaddem, M. A. G. Elferink, M. C. Seeleman, E. Dekker, C. J. A. Punt, O. Visser, M. L. Essink-Bot

**Affiliations:** 1 Department of Public Health, Academic Medical Center, Meibergdreef 9, 1105AZ Amsterdam, The Netherlands; 2 Netherlands Comprehensive Cancer Care Organisation, Utrecht, The Netherlands; 3 Department of Gastroenterology and Hepatology, Academic Medical Center, Amsterdam, The Netherlands; 4 Department of Medical Oncology, Academic Medical Center, Amsterdam, The Netherlands

**Keywords:** Colon cancer, Ethnic minorities, Health care utilisation, Quality of care, Register data

## Abstract

**Background:**

Ethnic differences in colon cancer (CC) care were shown in the United States, but results are not directly applicable to European countries due to fundamental healthcare system differences. This is the first study addressing ethnic differences in treatment and survival for CC in the Netherlands.

**Methods:**

Data of 101,882 patients diagnosed with CC in 1996–2011 were selected from the Netherlands Cancer Registry and linked to databases from Statistics Netherlands. Ethnic differences in lymph node (LN) evaluation, anastomotic leakage and adjuvant chemotherapy were analysed using stepwise logistic regression models. Stepwise Cox regression was used to examine the influence of ethnic differences in adjuvant chemotherapy on 5-year all-cause and colorectal cancer-specific survival.

**Results:**

Adequate LN evaluation was significantly more likely for patients from ‘other Western’ countries than for the Dutch (OR 1.09; 95% CI 1.01–1.16). ‘Other Western’ patients had a significantly higher risk of anastomotic leakage after resection (OR 1.24; 95% CI 1.05–1.47). Patients of Moroccan origin were significantly less likely to receive adjuvant chemotherapy (OR 0.27; 95% CI 0.13–0.59). Ethnic differences were not fully explained by differences in socioeconomic and hospital-related characteristics. The higher 5-year all-cause mortality of Moroccan patients (HR 1.64; 95% CI 1.03–2.61) was statistically explained by differences in adjuvant chemotherapy receipt.

**Conclusion:**

These results suggest the presence of ethnic inequalities in CC care in the Netherlands. We recommend further analysis of the role of comorbidity, communication in patient-provider interaction and patients’ health literacy when looking at ethnic differences in treatment for CC.

## Background

Colon cancer (CC) is one of the most frequent cancers in Europe, and the second most frequent cause of cancer death in the Netherlands [1]. In 2014, the age-standardized incidence rate of CC was 28.4 per 100,000 (World Standardized Population), comparable to that of other Western countries [2].

Diagnosis and treatment of CC are framed in a well-documented, evidence-based set of guidelines, developed by a multidisciplinary group of specialists. The primary treatment for non-metastatic CC is surgery. After the operation, adjuvant chemotherapy should be considered for patients with lymph node metastases upon pathologic assessment of the resection specimen. Therefore, adequate lymph node evaluation is important [3].

Research in the Netherlands has shown a generally improving quality of CC care over time [4]. However, no specific attention has been paid to differences in treatment of CC across ethnic groups in the Netherlands, while several studies showed ethnic differences in health care utilization in the country [5]. Studies from the USA [6–8] show a lesser accessibility of screening care for ethnic minority groups [9], a lower use of adjuvant chemotherapy [10] and a lower rate of adequate lymph node evaluation [11]. These ethnic differences in accessibility and quality of care cannot be fully attributed to the generally lower socioeconomic position of ethnic minority patients. Geographical concentration of ethnic minority patients in some hospitals might contribute to differences in quality of CC care. A Dutch study showed variation across hospital types in treatment and outcome of patients with CC [12].

However, the USA situation is not fully comparable to the Dutch situation, due to fundamental differences in health system organisation, screening policies (no population-based screening for CC in the Netherlands until 2014), insurance coverage and their impact on ethnic and socioeconomic differences in access to health care.

Therefore, the present study investigates ethnic differences in CC care in the Netherlands, and their impact on survival. The research questions are: 1) Are there ethnic differences in guideline-recommended care for patients with colon cancer?; 2) Are ethnic differences in CC care attributable to groups differences in socioeconomic factors and hospital characteristics?; 3) Do differences in CC care affect ethnic differences in survival?

## Methods

### Theoretical framework

**Fig. 1:**
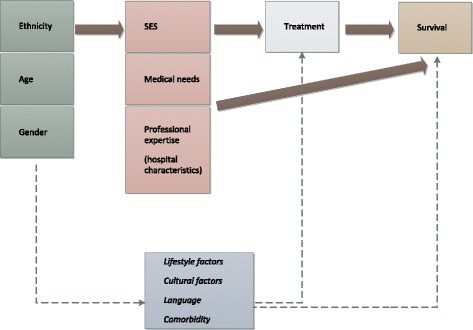
Theoretical framework Ethnic differences in CC treatment and survival

The analyses were framed in a conceptual framework adapted from the Behavioral model of access to medical care [13] and the Equity framework [5]. According to these, use of healthcare does not only result from medical need, but is also influenced by patients’ socioeconomic and cultural factors. These vary by ethnicity and may therefore (partly) explain ethnic variations in CC care. Equitable access to medical care implies that medical need is the key determinant for healthcare use, and that socioeconomic and cultural aspects influence health care use as little as possible. Therefore, ethnic origin should also ideally not influence the use of healthcare, unless it directly relates to the medical need, or influences the outcomes of care [5]. Figure 1 presents the relationships studied or accounted for in the present paper (bold arrows). The dashed lines represent other relationships hypothetically at play in ethnic differences in CC treatment and survival, but not investigated within this paper.

### Data and inclusion

Data were extracted from the Netherlands Cancer Registry (NCR) [1], a nationwide population-based registry including all newly diagnosed malignancies. Main sources of notification are the automated pathology archive (PALGA) and the Hospital Discharge Register (HDR). NCR data on patient characteristics, tumor characteristics and treatment are collected from hospital patient files by specially trained registration clerks and coded according to a national manual. Topography and morphology are coded according to the International Classification of Diseases for Oncology (ICD-O) and stage according to the TNM classification [14, 15]. Data quality is high and completeness is estimated to be at least 95% [16, 17].

For this study, data on all CC patients diagnosed between 1996 and 2011 were probabilistically linked (using date of birth, gender and postal code) to three databases of Statistics Netherlands (CBS) at individual level: the Dutch population register, the causes of death register and the social statistics database. Of all records in the NCR, 98.2% were successfully matched to the CBS databases. Information on the quality and completeness of the databases of Statistics Netherlands can be found elsewhere [18].

### Explanatory variables and confounders

#### Ethnic origin, age, gender

Ethnic origin (based on country of birth of the patient and his/her parents) was obtained from the CBS population register. Categories were Dutch, other Western, Turkish, Moroccan, Surinamese, Antillean and other non-Western, using the definition established by CBS [19]. Age at diagnosis and gender were obtained from the NCR.

#### Socioeconomic status (SES)

We used type of employment/main income source as SES indicator. Data was obtained from the CBS social statistics database and categorized into 8 modalities: employed, self-employed, managing director, other active, unemployed (living from unemployment benefit, social welfare provision or other social welfare benefit), sickness leave, retired, and all others persons without income.

#### Medical need

Medical need was approximated by tumor site (right-sided (C18.0-C18.2), transverse (C18.3-C18.5), left-sided (C18.6-C18.7) and overlapping lesions or not otherwise specified (C18.8-C18.9)) and stage (I, II, III, IV) at diagnosis. All data were obtained from the NCR.

#### Hospital characteristics

Professional expertise was approximated using NCR data on hospital type (general, university and STZ, which are non-academic teaching hospitals) and hospital volume (low volume: <50, medium volume: 50–100, and high volume: >100 colon resections per year). Information on the region of hospital was also used in the analyses, to adjust for regional differences in concentration of ethnic minorities.

### Outcome variables

We used adequate lymph node (LN) evaluation, adjuvant chemotherapy and anastomotic leakage as CC care quality indicators. The choice for these indicators was based on current guidelines [3] and on the availability of data. All indicators were retrieved from the NCR.

Survival was operationalized as 5-years mortality (all-cause and colorectal cancer-specific, respectively). Since accuracy of cause of death is limited for colon and rectal cancer separately [20, 21], we used colorectal cancer-specific mortality. Colorectal cancer specific mortality was retrieved from the Cause of Death Register (CBS) (main primary cause ICD −10 codes C18 through C20).

### Statistical analyses

#### Treatment

Multivariate logistic regression analyses were performed to examine the association between ethnicity and adequate LN evaluation, defined as 10 or more evaluated LNs, among patients with stage I-III disease diagnosed in the period 1999–2011 and who underwent surgery. Variables on 1) age, gender, medical need, 2) hospital characteristics and 3) SES were entered stepwise into the model, to explore the influence of these (combinations of) factors on ethnic differences in adequate LN evaluation. Similar analyses were performed to analyse the association between ethnicity and adjuvant chemotherapy among patients with stage III disease in 1996–2011 who underwent surgery. Likewise, the association between ethnicity and risk of anastomotic leakage among patients who underwent surgery (2008–2011) was examined stepwise.

#### Survival

We used Cox proportional hazard models to investigate the influence of ethnic differences in adjuvant chemotherapy on survival. These models were built stepwise, introducing in the last step the receipt of adjuvant chemotherapy, to examine the impact of ethnic differences in the receipt of adjuvant chemotherapy on survival. Follow-up time was calculated as the time from the CC diagnosis to the end of follow-up or death (all causes or colorectal cancer death).

All multivariate analyses were adjusted for the year of diagnosis. Data were analysed using SPSS, version 13.0.

## Results

Table 1 displays patients’ characteristics: Dutch patients accounted for 89.6%, other Western patients for 8.5% and non-western patients for 1.9% of the study population.

**Table 1:** Characteristics of the study population per ethnic group, all patients diagnosed with colon cancer in The Netherlands from 1996 to 2011. *N* = 101,882, n (%)

		Western		Non-Western				
		The Netherlands 91,257 (89.6)	Other Western 8668 (8.5)	Turkey 289 (0.3)	Morocco 227 (0.2)	Suriname 693 (0.7)	Netherlands Antilles 236 (0.2)	Other non-Western 512 (0.5)
Gender	Male	45,598 (50.0)	4292 (49.6)	164 (56.7)	149 (65.6)	328 (47.3)	107 (45.3)	283 (55.3)
	Female	45,659 (50.0)	4370 (50.4)	125 (43.3)	78 (34.4)	365 (52.7)	129 (54.7)	229 (44.7)
Occupation (at diagnosis) (*n* = 96,902)	Employed^a^	11,598 (12.7)	1070 (12.3)	52 (18.0)	34 (15.0)	178 (25.7)	63 (26.7)	111 (21.7)
	Unemployed^b^	9077 (9.9)	1229 (14.2)	117 (40.5)	91 (40.1)	178 (25.7)	83 (35.2)	221 (43.2)
	Retired	66,066 (72.4)	5962 (68.8)	116 (40.1)	95 (41.9)	313 (45.2)	85 (36.0)	163 (71.5)
	Unknown	4516 (4.9)	407 (4.7)	4 (1.4)	7 (3.1)	24 (3.5)	5 (2.1)	17 (3.3)
Age (at diagnosis)	<60 yrs	15,318 (16.8)	1505 (17.4)	138 (47.8)	106 (46.7)	283 (40.8)	107 (45.3)	254 (49.6)
	60–74 yrs	38,005 (41.6)	4088 (47.2)	128 (44.3)	106 (46.7)	290 (41.8)	94 (39.8)	187 (36.5)
	>74 yrs.	37,934 (41.6)	3075 (35.5)	23 (8.0)	15 (6.6)	120 (17.3)	35 (14.8)	71 (13.9)
Stage (at diagnosis)	I	13,510 (14.8)	1265 (14.6)	38 (13.1)	17 (7.5)	99 (14.3)	33 (14.0)	69 (13.5)
	II	31,002 (34.0)	2988 (34.1)	115 (39.8)	75 (33.0)	206 (29.7)	81 (34.3)	149 (29.1)
	III	23,710 (26.0)	2248 (26.0)	69 (23.9)	68 (30.0)	212 (30.6)	60 (25.4)	167 (32.6)
	IV	20,523 (22.5)	1962 (22.7)	61 (21.1)	63 (27.8)	155 (22.4)	58 (24.6)	116 (22.7)
	Unknown	2379 (2.6)	224 (2.6)	6 (2.1)	4 (1.8)	21 (3.0)	4 (1.7)	11 (2.1)
Surgery	None	11,391 (12.5)	1066 (12.3)	27 (9.3)	32 (14.1)	81 (11.7)	26 (11.0)	50 (9.8)
	Endoscopic resection	2318 (2.5)	210 (2.4)	7 (2.4)	4 (1.8)	17 (2.5)	5 (2.1)	12 (2.3)
	Surgical resection	77,548 (85.0)	7392 (85.3)	255 (88.2)	191 (84.1)	595 (85.9)	205 (86.9)	450 (87.9)

^a^employee, managing director, self-employed, other active ^b^unemployment benefit, sickness benefit, other without income

The ethnic differences in gender, type of main occupation/income, age and stage at diagnosis were statistically significant (*p* < 0.05).

### Treatment

‘Other Western’ and all groups of non-Western stage I-III patients were more likely to receive adequate LN evaluation than Dutch patients. These differences were statistically significant for the ‘other Western’, the Turkish, the Moroccan and the Surinamese groups (Model A, Table 2). After adjusting for age, gender and medical need, the proportion of patients who received adequate LN evaluation was statistically significantly higher only for the ‘other Western’ group than for the Dutch reference group (Model B). Adjusting analyses for SES and/or hospital characteristics did not further explain the observed ethnic differences in adequate LN evaluation (Models C-E).

**Table 2:** Stepwise regression coefficients (ORs) for LN evaluation > = 10 for Stage I-III colon cancer patients (incidence year 1999–2011). *N* = 57,559

	> = 10 LNs	Model A: Univariate		Model B: Adjusted for age, gender and medical need^a^		Model C: Adjusted for age, gender, medical need^a^ and SES		Model D: Adjusted for age, gender, medical need^a^ and hospital characteristics		Model E: Adjusted for age, gender, medical need^a^, hospital characteristics^b^ and SES	
	%	OR	95% CI	OR	95% CI	OR	95% CI	OR	95% CI	OR	95% CI
Dutch	56.4	1.00		1.00		1.00		1.00		1.00	
Other Western	58.8	1.11	1.04–1.17	1.08	1.00–1.51	1.08	1.01–1.15	1.09	1.01–1.16	1.09	1.01–1.16
Turkey	64.0	1.38	1.02–1.87	0.79	0.56–1.11	0.80	0.57–1.12	0.81	0.58–1.15	0.83	0.59–1.16
Morocco	71.2	1.91	1.30–2.82	0.91	0.59–1.38	0.91	0.60–1.39	0.88	0.57–1.36	0.89	0.58–1.36
Suriname	64.8	1.43	1.17–1.75	1.25	0.99–1.56	1.25	1.00–1.57	1.15	0.91–1.44	1.15	0.92–1.45
Netherlands Antilles	60.4	1.18	0.84–1.66	0.77	0.52–1.13	0.77	0.52–1.13	0.74	0.50–1.09	0.74	0.50–1.09
Other non-Western	61.8	1.25	0.98–1.59	0.89	0.68–1.17	0.90	0.68–1.18	0.83	0.62–1.09	0.83	0.63–1.09

^a^Tumor site and stage at diagnosis

^b^Hospital type, hospital volume and region

In the univariate analyses (Model A, Table 3), stage III patients of all other ethnic origins, except the Moroccan group, were significantly more likely to receive adjuvant chemotherapy than the . Multivariate models (Models B, C, D, E) showed that patients of Moroccan and Surinamese origin were significantly less likely than Dutch patients to receive adjuvant chemotherapy. Adding and exchanging SES and hospital characteristics had little effect on the observed results.

**Table 3:** Stepwise regression coefficients (ORs) for adjuvant chemotherapy for Stage III colon cancer patients (incidence year 1996–2011). *N* = 22,063

	Adjuvant chemotherapy	Model A: Univariate		Model B: Adjusted for age, gender and medical need^a^		Model C: Adjusted for age, gender, medical need^a^ and SES		Model D: Adjusted for age, gender, medical need^a^ and hospital characteristics^b^		Model E: Adjusted for age, gender, medical need^a^, hospital characteristics^b^ and SES	
	%	OR	95% CI	OR	95% CI	OR	95% CI	OR	95% CI	OR	95% CI
Dutch	54.3	1.00		1.00		1.00		1.00		1.00	
Other Western	58.1	1.17	1.06–1.29	1.03	0.91–1.16	1.05	0.93–1.19	1.00	0.89–1.13	1.02	0.90–1.15
Turkey	75.4	2.59	1.41–4.73	0.51	0.254–1.04	0.56	0.28–1.12	0.54	0.27–1.09	0.60	0.30–1.19
Morocco	66.7	1.68	0.92–3.071	0.26	0.12–0.57	0.28	0.13–0.59	0.26	0.12–0.57	0.27	0.13–0.59
Suriname	66.7	1.68	1.23–2.31	0.54	0.36–0.81	0.60	0.40–0.89	0.61	0.40–0.92	0.69	0.45–1.00
Netherlands Antilles	71.7	2.14	1.12–4.06	0.56	0.26–1.20	0.81	0.37–1.76	0.60	0.28–1.23	0.85	0.39–1.86
Other non-Western	75.8	2.64	1.75–3.98	0.69	0.41–1.17	0.90	0.53–1.54	0.75	0.44–1.28	0.98	0.57–1.68

^a^Tumor site and stage at diagnosis

^b^Hospital type, hospital volume and region

‘Other Western’ and ‘other non-Western’ patients were significantly more likely than Dutch patients to experience anastomotic leakage after surgery (Table 4, Model A). In all other models, only the ‘other Western’ group was significantly more at risk for anastomotic leakage than the Dutch. Adding hospital characteristics, as in Model D, seemed to have an explanatory effect similar to the one of SES (Model C) on reducing ethnic differences in point estimates.

**Table 4:** Stepwise regression coefficients (ORs) for anastomotic leakage for colon cancer patients with surgical resection (incidence year 2008–2011). *N* = 24,620

	Anastomotic leakage	Model A: Univariate		Model B: Adjusted for age, gender and medical need^a^		Model C: Adjusted for age, gender, medical need^a^ and SES		Model D: Adjusted for age, gender, medical need^a^ and hospital characteristics^b^		Model E: Adjusted for age, gender, medical need^a^, hospital characteristics^b^ and SES	
	%	OR	95% CI	OR	95% CI	OR	95% CI	OR	95% CI	OR	95% CI
Dutch	6.3	1.00		1.00		1.00		1.00		1.00	
Other Western	8.1	1.30	1.10–1.53	1.29	1.09–1.53	1.29	1.09–1.53	1.24	1.05–1.47	1.24	1.05–1.47
Turkey	8.2	1.37	0.66–2.84	1.28	0.62–2.67	1.21	0.58–2.51	1.29	0.62–2.69	1.22	0.58–2.55
Morocco	8.5	1.40	0.64–3.04	1.24	0.57–2.71	1.17	0.54–2.58	1.27	0.58–2.78	1.20	0.55–2.64
Suriname	5.5	0.87	0.46–1.66	0.84	0.44–1.60	0.79	0.42–1.51	0.83	0.43–1.58	0.78	0.41–1.49
Netherlands Antilles	8.5	1.40	0.64–3.04	1.37	0.63–2.99	1.28	0.58–2.80	1.36	0.62–2.98	1.27	0.58–2.78
Other non-Western	10.8	1.72	1.00–2.94	1.63	0.95–2.80	1.51	0.87–2.61	1.65	0.96–2.85	1.53	0.88–2.65

^a^Tumor site and stage at diagnosis

^b^Hospital type, hospital volume and region

### Mortality

Table 5 displays ethnic differences in CRC-specific mortality. In the first model, the ‘other Western’ group had lower 5-year hazard ratios (HRs) than the Dutch reference group (Model A, univariate). In Model B, adjusting for age, gender, medical need, SES and hospital characteristics, the lower HRs of the non-Western group subsisted. Adding the variable ‘adjuvant chemotherapy’ to the analyses (Model C) to examine the impact of differences in adjuvant chemotherapy on differences in survival between groups, did not change this. However, the HRs of the Moroccan group showed a noticeable decrease when adding information on adjuvant chemotherapy.

**Table 5:** 5-year cause-specific mortality (HRs) by ethnicity for Stage III colon cancer patients who underwent surgery (incidence year 1996–2011). *N* = 18,214

	Model A: Univariate		Model B: Adjusted for age, gender, medical need^a^, hospital characteristics^b^ and SES		Model C: Adjusted for age, gender, medical need^a^, hospital characteristics^b^, SES and adjuvant chemotherapy	
	HR	95% CI	HR	95% CI	HR	95% CI
Dutch	1.00		1.00		1.00	
Other Western	0.87	0.78–0.96	0.88	0.79–0.98	0.87	0.78–0.97
Turkey	0.74	0.41–1.35	0.88	0.48–1.59	0.87	0.48–1.57
Morocco	0.73	0.38–1.40	1.19	0.62–2.30	1.01	0.52–1.94
Suriname	0.92	0.68–1.22	1.20	0.90–1.61	1.14	0.85–1.53
Netherlands Antilles	0.83	0.46–1.51	1.26	0.69–2.28	1.23	0.68–2.24
Other non-Western	0.80	0.55–1.18	1.16	0.79–1.72	1.16	0.79–1.72

^a^Tumor site and stage at diagnosis

^b^Hospital type, hospital volume and region

All-cause mortality rates was significantly higher among Moroccan and ‘other non-Western’ origin stage III patients who underwent surgery than among the Dutch, after adjustment for age, sex and medical need (Model B). Addition of the variable ‘adjuvant chemotherapy’ to the analyses (Model C), to examine the impact of differences in adjuvant chemotherapy to differences in survival, the HRs of the Moroccan group dropped under significance level, while the higher HRs of the ‘other non-Western group’ remained unchanged (Table 6).

**Table 6:** 5-year all-cause mortality (HRs) by ethnicity for Stage III colon cancer patients who underwent surgery (incidence year 1996–2011). *N* = 18,214

	Model A: Univariate		Model B: Adjusted for age, gender, medical need^a^, hospital characteristics^b^ and SES		Model C: Adjusted for age, gender, medical need^a^, hospital characteristics^b^, SES and adjuvant chemotherapy	
	HR	95% CI	HR	95% CI	HR	95% CI
Dutch	1.00		1.00		1.00	
Other Western	0.94	0.87–1.02	0.96	0.89–1.05	0.96	0.89–1.04
Turkey	0.74	0.46–1.17	0.97	0.61–1.54	0.97	0.61–1.54
Morocco	0.89	0.56–1.41	1.64	1.03–2.61	1.34	0.84–2.13
Suriname	0.81	0.64–1.03	1.15	0.90–1.47	1.08	0.85–1.38
Netherlands Antilles	0.78	0.49–1.26	1.27	0.78–2.04	1.23	0.77–1.99
Other non-Western	0.86	0.65–1.15	1.34	1.00–1.80	1.35	1.01–1.03

^a^Tumor site and stage at diagnosis

^b^Hospital type, hospital volume and region

## Discussion

This nationwide study showed ethnic differences for three indicators of CC care, which could not be fully explained by differences in socioeconomic and hospital-related characteristics. Adequate LN evaluation was more likely to take place for patients from other Western countries, compared to the Dutch. However, this same group had also a slightly higher risk of anastomotic leakage after surgical resection. Adjuvant chemotherapy was less likely to be administered to Moroccan than to the Dutch, and this was not fully explained by differences in socioeconomic factors and hospital characteristics. Survival analyses did not show a significant effect of the observed ethnic differences in receipt of adjuvant chemotherapy on CRC-specific mortality. However, the lower receipt of adjuvant chemotherapy in the Moroccan group was significantly associated with a higher all-cause mortality rate.

This outcome was not found in the 5-year colorectal cancer mortality analyses. This could point at another explanation: in the all-cause survival analyses, adjuvant chemotherapy might be a marker for comorbidity, and therefore explain the excess all-cause mortality of the Moroccan group. Information on comorbidity was not available in our study, but the Moroccan group is known to suffer more often from chronic diseases like diabetes [22] and hypertension [23]. Noticeably, CRC patients with diabetes have been shown to receive less often chemotherapy than non-diabetes patients [24]. Therefore, the effect of adjuvant chemotherapy on the excess mortality rate of the Moroccan CC patients in our study might be standing for a higher comorbidity within this group, which would explain the higher 5-year all-cause mortality, without having any significant effect on the 5-year colorectal cancer-specific mortality. The lack of comorbidity data in the NCR limited the interpretation of the present analyses.

Also, information on the presence and treatment of metachronous metastases was not available for this study, while this factor also influences all-cause survival. Other indicators of need for CC care were however taken into account, like stage, tumour localisation and differentiation.

Other factors suggested in the literature to be at play in ethnic differences in CC care include differences in insurance coverage [5, 6] and difficulties in patient-provider interaction. Different insurance coverage does not form a likely explanation in universal access healthcare systems as in most European countries. However, a language barrier, lower patient health literacy or cultural differences may complicate the patient-provider interaction, and challenge the quality of care provided. A decision about adjuvant chemotherapy requires patient involvement, and the above-mentioned difficulties might explain the lower receipt of adjuvant chemotherapy for the Moroccan group. The similarity in LN evaluation and anastomotic leakage between the Dutch and all non-Western groups involved is also in accordance with this thought, these quality indicators being less related to the patient active involvement in the decision-making process. However, the increased likelihood of LN evaluation and anastomotic leakage among ‘other Western’ patients remains unexplained. Morphological or anatomic differences between ‘other Western’ patients and the Dutch patients might explain parts of the observed differences.

The relatively low numbers in the non-Western ethnic minority groups limited the statistical power of the analyses for the present study. The numbers are in accordance with expectations, given the low incidence of colon cancer in these relatively young groups. Nevertheless, this study included all CC patients diagnosed in the Netherlands over a period of 15 years. Linking NCR data to the Dutch population register made the ethnic categorization possible, and linkage with the causes of death register and the social statistics database enabled further analyses and model adjustments.

Future research should primarily focus on the role of comorbidity. Registries offer an outstanding opportunity to investigate groups differences in guideline-recommended care. However, the lack of information on comorbidity within the NCR limits the scope of conclusions, and should therefore be tackled. International research showed the importance of this information when studying ethnic differences in CC survival [25].

The role of communication and health literacy also deserves attention when looking at ethnic differences in treatment for CC. The interaction between physician and patients should be investigated specifically, and research should deepen the motivations of physicians when deciding upon treatment in an ethnically-diverse patient population. Qualitative techniques might be a very appropriate choice when unravelling the mechanisms at play in this interaction.

## Conclusion

In conclusion, ethnicity should be taken into account when assessing quality of CC care in the Netherlands and elsewhere. Important differences are present, which cannot be solely attributed to tumor stage, age and gender differences, or differences between hospitals, and therefore point at ethnic inequities in quality of CC care. This is all the more important as ethnic inequalities in guideline-recommended care might also lead to ethnic inequalities in mortality, as shown in this study’s results.

## Abbreviations

CBS: Statistics Netherlands
CC: Colon cancer
CRC: Colorectal cancer
HDR: Hospital discharge register
HR: Hazard ratio
ICD-O: International classification of diseases for oncology
LN: Lymph node
NCR: Netherlands cancer registry
OR: Odd ratio
PALGA: Automated pathology archives
SES: Socio-economic status
